# Stable Heteroclinic Channel Networks for Physical Human–Humanoid Robot Collaboration

**DOI:** 10.3390/s23031396

**Published:** 2023-01-26

**Authors:** Tilen Brecelj, Tadej Petrič

**Affiliations:** Department of Automatics, Biocybernetics and Robotics, Jožef Stefan Institute, Jamova Cesta 39, 1000 Ljubljana, Slovenia

**Keywords:** human–robot interaction, collaborative robots, phase state system, stable heteroclinic channel networks

## Abstract

Human–robot collaboration is one of the most challenging fields in robotics, as robots must understand human intentions and suitably cooperate with them in the given circumstances. But although this is one of the most investigated research areas in robotics, it is still in its infancy. In this paper, human–robot collaboration is addressed by applying a phase state system, guided by stable heteroclinic channel networks, to a humanoid robot. The base mathematical model is first defined and illustrated on a simple three-state system. Further on, an eight-state system is applied to a humanoid robot to guide it and make it perform different movements according to the forces exerted on its grippers. The movements presented in this paper are squatting, standing up, and walking forwards and backward, while the motion velocity depends on the magnitude of the applied forces. The method presented in this paper proves to be a suitable way of controlling robots by means of physical human-robot interaction. As the phase state system and the robot movements can both be further extended to make the robot execute many other tasks, the proposed method seems to provide a promising way for further investigation and realization of physical human–robot interaction.

## 1. Introduction

Everyday movements such as sitting, standing, running, jumping, handling and manipulating different objects, and many others are fairly simple for healthy humans to perform. Furthermore, there are many more complicated tasks that humans perform on daily basis in collaboration with other humans, such as dancing, carrying different objects, or pushing and pulling while holding each other to provide help and assistance for unhealthy or disabled individuals. While physically interacting with each other, humans can easily understand mutual intentions and can adapt instantaneously to the other person’s behavior to suitably cooperate and provide the necessary assistance. This is possible because humans are capable of interpreting body motion and different gestures typical for human non-verbal communication [1,2]. Moreover, if speech is added to the communication between humans, cooperation becomes even smoother.

While performing all possible tasks, humans can subconsciously coordinate their bodies and take care of their stability to fully control their motion. In addition, while physically interacting with other individuals, humans can not only mutually adapt to their collaborators but also take care of his/her stability. This allows humans to smoothly cooperate and complete different kinds of tasks even if no prior agreement on the course of the task alone was made.

To assist humans, robots are being employed in different fields of industry and science [3,4], healthcare and medicine [5,6,7], hospitality and tourism [8,9,10], as domestic robots [11] and in many other areas. For such applications, humanoid robots are of special interest as they have similar body shapes as humans and could, therefore, learn to cooperate with them by mimicking human-like behavior. However, their disadvantage is their relatively small support polygon in comparison to their large body size, which makes them easily unstable when fast movements are performed, or external forces are applied, as is the case in physical human–robot interaction. This is why every movement or physical interaction with the environment represents a challenge for them to remain stable and not fall. To ensure stability to humanoid and other floating-based robots, i.e., robots that are not fixed to a surface, while performing different types of movements and physically interacting with the environment, various methods and stability parameters mostly based on the zero moment point [12,13,14,15,16,17,18,19,20,21,22], but also on the linear and angular momenta of the robot [23,24,25], gravito-inertial wrench cones for non-coplanar contacts [26,27], stability polygons [28], or model predictive control [29], have been developed. This enables an enormous advance not only in the development of humanoid robot motion techniques such as walking [30,31,32,33,34], running and jumping [35], skiing [36], and many others, but also in the development of skills for physical cooperation with humans [37], object grasping and manipulation [38,39,40,41,42,43] and other types of physical interaction with the environment. A review of the state-of-the-art advances in bipedal walking robots and their hardware design can be found in [44,45].

Robots mostly perform tasks on their own, and their physical cooperation with humans is very limited. Furthermore, most of the tasks performed by the robots are pre-programmed and pre-defined in advance; some robots are teleoperated by humans in real-time, although reactive control algorithms are also being extensively developed. However, robots do not have the feeling of mutual physical interaction with humans to keep each other balanced and optimize the interaction in a manner to assist humans in the best possible way. This is why physical collaboration tasks that robots can perform using the state-of-the-art reactive controllers are limited and very specific, as robots can not understand human intentions in an arbitrary scenario, and it is, therefore, impossible for them to assist humans in all everyday situations. It is, thus, crucial to study human behavior and motion to understand people’s intentions, as this dictates the course of the interaction [46,47,48]. Even though human–robot interaction and cooperation are still in their initial phases, it is one of the fastest-growing fields in robotics as different methods and strategies are being developed and applied to robots to make them understand human intentions and physically interact with them [49,50,51,52,53,54,55,56,57].

**Figure 1:**
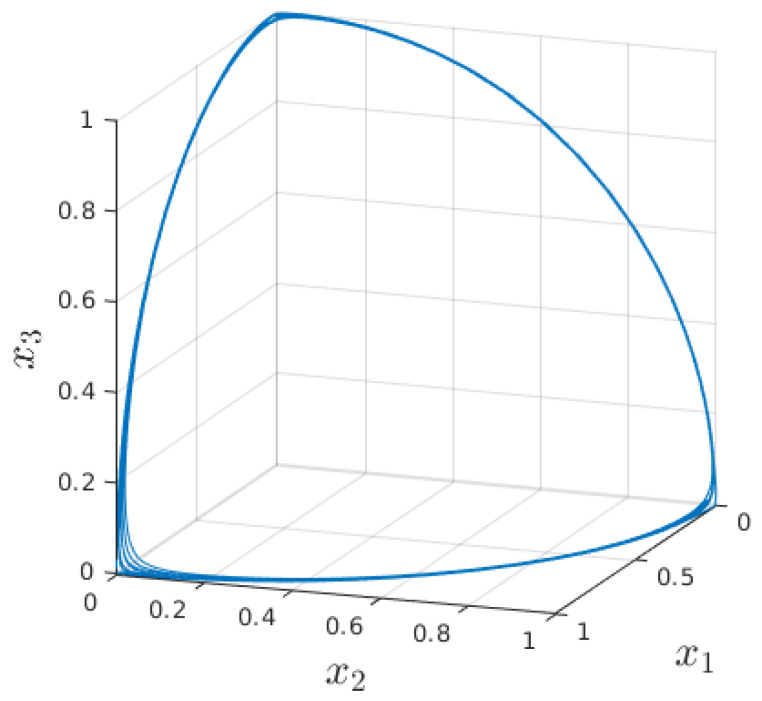
A representation of a PSS built on the SHC networks consisting of three states, each lying on its coordinate axis and connected with multiple SHC.

One way to make robots collaborate with humans is using the stable heteroclinic channel (SHC) networks [58,59,60,61]. This is a continuous dynamical phase state system (PSS) connecting an arbitrary number of saddle points that can be interpreted as states, as shown in Figure 1.

The transitions between them are smooth; they can be reversed, and most importantly, they have a phase. Such an SHC system can be coupled to different robot motions, where each state corresponds to a certain final robot configuration, while the robot motion from the initial to the final state is coupled to the phase of the SHC transition [62].

In this paper, first, the SHC networks are defined and presented on a simple model consisting of three states. Second, the SHC networks are implemented in a more complex dynamical model consisting of eight states. In the end, the eight-state SHC networks system is coupled to the motion of a humanoid robot, where the state transitions are triggered by the forces applied to the grippers of the humanoid robot. By pushing or pulling the grippers of the humanoid robot up or down, a person triggers different SHC state transitions and, this way, controls the motion of the robot. According to the SHC states, the humanoid robot stands still, squats, stands back up, and walks forwards or backwards.

With the coupling of the SHC networks to the robot motion, it is shown how this dynamical model can be used to guide robots and make them cooperate with humans. Although for the tasks presented in this paper, the robot motion was pre-defined, the investigated approach confirms to be a promising way for further development of human–robot collaboration techniques.

In Materials and Methods first, the SHC networks are defined and applied to a simple three-state PSS. Further on, the application of an eight-state PSS guided by the SHC networks to the motion of a humanoid robot is presented, and the robot motion synthesis is described. In Results, all the possible PSS transitions resulting in robot squatting, standing up, and walking forwards and backward are presented and described. In Discussion and Conclusions, the application of the presented PSS to a humanoid robot is validated in the means of human–robot interaction and cooperation, and the directions for further development are given.

## 2. Materials and Methods

### 2.1. The Definition of the SHC Networks

The method that controls the robot motion presented in this paper is built on the SHC networks [58,59,60,61]. This is a dynamical PSS that connects saddle points which can be interpreted as states, each lying on its coordinate axis. The transitions between states occur via self-stabilizing limit cycles, each with its own velocity, activation time, and phase, and can be arbitrarily allowed or prohibited. Furthermore, the transitions are nonexclusive, so more transitions can be initialized at the same time. In addition, the transitions can also be reversed, and the system returns to the initial state. A representation of a three-state SHC network is presented in Figure 1.

The dynamical PSS built on the SHC networks can be for a *n*-dimensional state vector $\vec{x}$ defined as

(1) $$\dot{\vec{x}}=\vec{x}\circ \left(\vec{\alpha }+\left(\rho_{0}+\rho_{\Delta }\circ \left(T+G\right)\right){\vec{x}}^{\gamma }\right)\eta \left(t\right)+\dot{\vec{\delta }}\left(t\right)\;,$$

where ∘ denotes element-wise multiplication [61]. The matrix T is the state transition matrix with values $T_{ji}=1$ if the transition i→j exists and $T_{ji}=0$ otherwise, while the matrix G is the greediness matrix which determines the probability of the system to maintain multiple transitions activated, but it can also gradually reverse a transition. The n×n matrix

(2) $$\rho_{0}=\left(\vec{\alpha }{\left({\vec{\beta }}^{-1}\right)}^{T}\right)\circ \left(I-1-{\left(\vec{\alpha }{\vec{\alpha }}^{T}\right)}^{-1}\right)$$

defines the saddle points, each located on its coordinate axis, while the matrix product $\rho_{\Delta }\circ T$ determines the stable heteroclinic channels. The matrix $\rho_{\Delta }$ is also of the dimensions n×n and is defined as

(3) $$\rho_{\Delta }=\left(\vec{\alpha }\circ \left(1+{\vec{\nu }}^{-1}\right)\right){\left({\vec{\beta }}^{-1}\right)}^{T}\;.$$

The three *n*-dimensional parameter vectors $\vec{\alpha }$, $\vec{\beta }$, and $\vec{\nu }$ determine the growth rates, the state’s positions, and the shapes of the saddle points, respectively. The matrices $\rho_{0}$ and $\rho_{\Delta }$ maintain constant values, while the matrices T and G can be modified during the evolution of the dynamical system. The scalars γ and η(t) determine the location of the channels and adjust the evolution rate of the dynamical system, respectively, while $\dot{\vec{\delta }}\left(t\right)$ determines when the system will leave a state and pushes the system away from the saddle points along a certain transition.

As the states lie on each coordinate axis, they form an orthonormal basis. Each transition determined by the SHC networks, therefore, lies on the plane extending among the coordinate axes containing its corresponding initial and final state. The activation function for all possible transitions that is invariant to the scaling of $\vec{x}$ and can have values from the interval [0,1] can, therefore, be determined as

(4) $$\Lambda^{t}=\frac{16\vec{x}{\vec{x}}^{T}\left|{\vec{x}}^{2}\right|}{{\left(\vec{x}1^{T}+1{\vec{x}}^{T}\right)}^{2}+{\left|\vec{x}\right|}^{2}}\circ T\;,$$

where 1 is an *n*-dimensional vector of ones. The state activations of the dynamical system can be computed from the residual of the transition activation function as

(5) $$\lambda^{s}=\frac{{\vec{x}}^{2}}{\sum {\vec{x}}^{2}}\left(1-\sum \Lambda^{t}\right)\;.$$

As the diagonal elements of the transition matrix $\Lambda^{t}$ are meaningless, an activation matrix Λ containing all the state transitions and activations, with the property $\sum \Lambda_{ji}=1$, can be determined as

(6) $$\Lambda_{ji}=\left\{\begin{array}{cc} \Lambda_{ji}^{t}\;; & \;j\neq i\\ \lambda_{i}^{s}\;; & \;j=i \end{array}\right.\;.$$

With regard to the fact that transitions determined by the SHC networks lie on planes defined by their corresponding predecessor and successor states, a phase with a value from the interval [0,1] can be assigned to each active transition from the state *i* to the state *j* as

(7) $$\Phi_{ji}=\frac{|{\vec{x}}_{j}|}{|{\vec{x}}_{j}\left|+\right|{\vec{x}}_{i}|}\;.$$

The phase has the value 0 when thy system is in its initial state and reaches the value 1 when the system evolves to its final state.

The transition of the system to the state *j* can be triggered by applying a small positive value to ${\dot{\delta }}_{j}\left(t\right)$. On the contrary, a small negative value applied to ${\dot{\delta }}_{j}\left(t\right)$ prohibits the corresponding transition from occurring. To determine the velocity biases for each transition, $\dot{\vec{\delta }}\left(t\right)$ can be defined as

(8) $$\dot{\vec{\delta }}\left(t\right)=\left(\Lambda \circ B\right)\vec{x}\;,$$

where each element $B_{ji}$ of the bias matrix B specifies the velocity bias of the transition i→j. If more than one velocity bias from a certain state has a positive value, multiple transitions will be triggered at once, but the attractor’s shape will make the system converge towards only one final state.

To enable a continuous modification of the transition velocity, the scaling factor η(t) can be expressed as

(9) $$\eta \left(t\right)=2^{A\sum_{ji}\Lambda_{ji}}\;.$$

The parameter *A* speeds up or slows down a transition relative to the speed determined by the vector $\vec{\alpha }$ and can be modified in every iteration of the PSS. For the default transition speed, the value of A should be set to 0.

### 2.2. A Three-State PSS Guided by the SHC Networks

**Figure 2:**
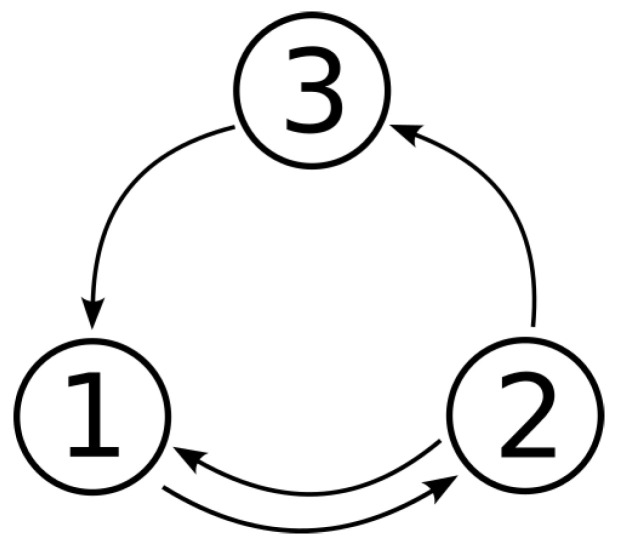
A schematic representation of a PSS guided by the SHC networks consisting of three states and the possible transitions between them.

For a better representation of how a PSS based on the SHC networks behaves, a simple three-state model is presented. The possible transitions between the states of the presented model are 1→2, 2→1, 2→3 and 3→1, as shown in Figure 2, so the state transition matrix is

(10) $$T=\left[\begin{array}{ccc} 0 & 1 & 1\\ 1 & 0 & 0\\ 0 & 1 & 0 \end{array}\right]\;.$$

All the elements of the greediness matrix G were set to 0 so that simultaneous multiple transitions were not started. The values of the vectors $\vec{\alpha }$, $\vec{\beta }$ and $\vec{\nu }$ were $\alpha_{i}=20$, $\beta_{i}=1$, and $\nu_{i}=1$, respectively, for i∈[1,3], which means that the growth rates and the saddle point shapes were all the same, while the saddle points were located each on its coordinate axis at the value 1. The parameter γ was set to 1, so the transitions directly left their corresponding initial state and approached their final state. In the beginning, the elements of the bias matrix were set to $B_{ji}=-10^{-4}$, so that all the transitions were blocked, while A was set to 0 so that the speed of the evolution of the system was no modified. Both the matrix B and the parameter A were modified during the simulation.

**Figure 3:**
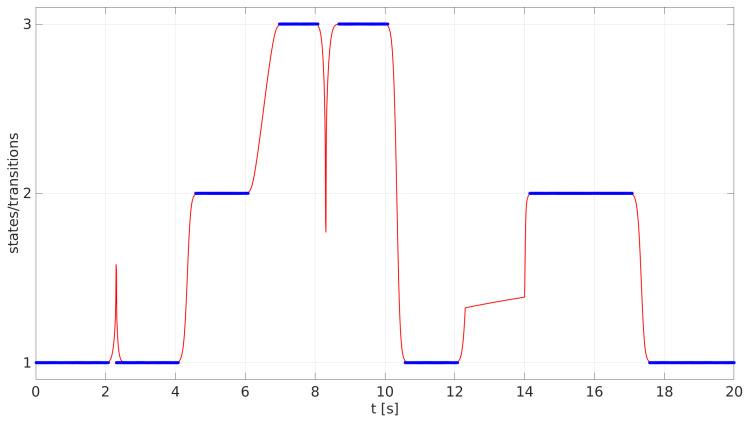
The time evolution of the three-state SHC system. The blue lines represent the system states, while the red lines represent the transitions from the initial to the final states and were obtained from the evolution phases from Equation (7).

The time evolution of the three-state SHC system is shown in Figure 3, where the step size of the model integration was set to $dt=10^{-2}$. The initial state of the system was set to 1 and, therefore, $\vec{x}\left(t=0\right)=\left(1,0,0\right)$. At the time t=2s, the bias matrix B was modified so that $B_{21}=10^{-1}$ and, therefore, the system transition to the state 2 was triggered. But at the time t=2.2s, the bias matrix B was modified so that $B_{12}=10^{3}$. With this modification, the system was forced to return to state 1 immediately. After returning to state 1, all the elements of the velocity bias matrix were set to the initial value $B_{ji}=-10^{-4}$, so the system was forced to stay in state 1.

At the time t=4s, the bias matrix element for the transition from state 1 to state 2 was again modified to $B_{21}=10^{-1}$ and, therefore, the transition of the system towards the state 2 resumed. When the system reached the final state, the state vector became $\vec{x}=\left(0,1,0\right)$.

At t=6s, the system was pushed towards the state 3, so all the elements of the bias matrix B were set to $-10^{-4}$, except $B_{32}=10^{-1}$. Furthermore, to decelerate the speed of the transition, the modification A=−10 was made. As can be seen in Figure 3, this transition between states 2 and 3 was slower compared to the previous transition from states 1 and 2. When the system reached state 3, the state vector became $\vec{x}=\left(0,0,1\right)$ and bot matrices A and B were set to the initial values.

At t=8s, the system was sent towards the state 1 by setting $B_{13}=10^{-1}$. After 0.2 s, the system was sent back to state 3 by setting $B_{31}=10^{3}$. When the system was back at state 3, all the elements of B were again set to the initial values so that the system remained in the current state.

The next transition occurred at time t=10s, when the system transited to state 1 by setting $B_{13}=10^{-1}$. At the end of the transition, the state vector became $\vec{x}=\left(1,0,0\right)$ and all the elements of the matrix B were again set to $-10^{-4}$ to fix the system in the current state.

At t=12s, the transition towards the state 2 was activated by setting $B_{21}=10^{-1}$. However, 0.03 s after its activation, the velocity of the transition was decreased by setting A=−50. At the time t=14s, the velocity of the transition was modified again; this time by increasing it relative to the reference velocity with A=10. The modifications in the transition velocities can be seen in Figure 3. When the system reached state 2, the state vector become $\vec{x}=\left(0,1,0\right)$ and both matrices A and B were set to their initial values.

Lastly, at t=17s, the value $B_{12}=10^{-1}$ was applied, and the system transited one more time to the state 1, and the state vector once again became $\vec{x}=\left(1,0,0\right)$.

Figure 3 shows the time evolution of the discussed PSS controlled by the SHC networks as it was guided by the parameters that control its behavior. The system transits between all three states according to the transitions allowed by the matrix T. Furthermore, some transitions were also reversed during their course. In addition, the velocities of the transitions were also modified before and during the transitions.

### 2.3. Application of the PSS Guided by the SHC Networks to the Humanoid Robot

Due to its maneuverability, a dynamical PSS controlled by the SHC networks is a suitable model for guiding robotic systems and controlling their motion. During the transition from an initial to a final state, the phase of the corresponding PSS transition, defined by Equation (7), increases from 0 to 1. If the robot motion is parametrized, it can be coupled to the phase of the PSS, and this way, the robot moves according to the phase of the corresponding PSS transition. When the phase of the PSS transition is 0, the robot is in its initial state, as it is the PSS itself. However, as the phase of the PSS is modified, the robot modifies its configuration and finally reaches its final gait when the phase of the transition is 1, and the system reaches its final state.

#### 2.3.1. The Motion Tasks and Their Corresponding Movements

**Figure 4:**
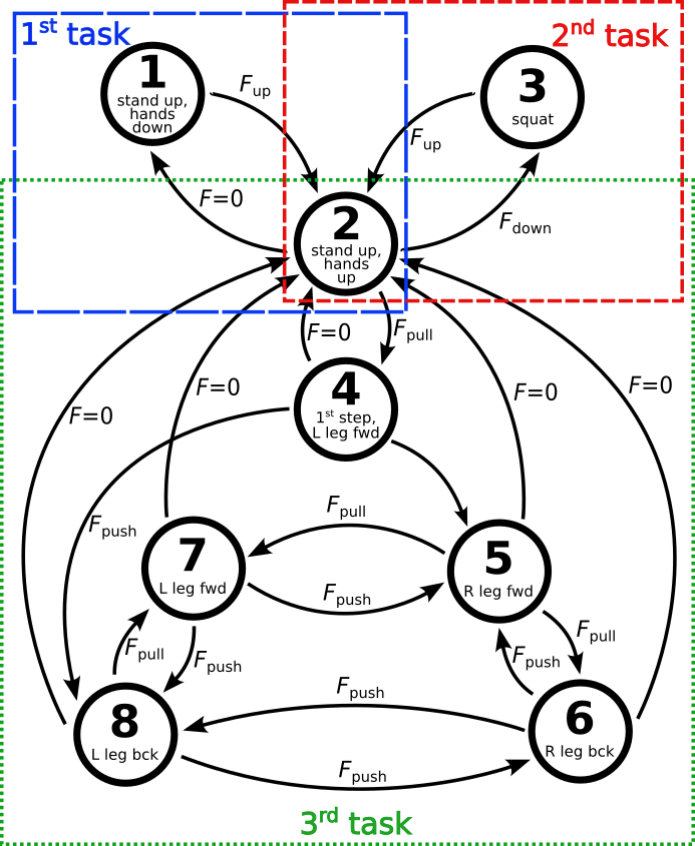
A schematic representation of the dynamical PSS consisting of 8 states, guided by the SHC networks, with the corresponding positions of the humanoid robot and the conditions for the transitions between the PSS states to occur. The numbered circles represent the PSS states with the corresponding humanoid robot positions. The arrows between the circles represent the possible PSS transitions, while the forces next to the arrows are the conditions that trigger the corresponding transition. The blue (dashed), red (finely dashed), and green (dotted) rectangles encompass the PSS states, the transitions between them, and the configurations of the humanoid robot for the first, second, and third motion task, respectively.

In this paper, the dynamical PSS controlled by the SHC networks is employed to guide the humanoid robot Talos with 32 degrees of freedom that can perform human-like motion [63]. The robot movements presented in this paper constitute three different tasks, as shown in Figure 4.

Each state of the PSS is connected to a specific robot configuration, while the transitions between the PSS states, which cause the robot motion and the execution of a certain task, are triggered by the forces applied to the wrist grippers of the humanoid robot, measured by the force-torque sensors mounted in the grippers.

**Figure 5:**
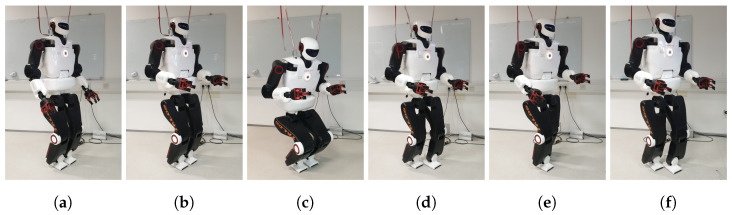
(**a**–**f**) Photos of the humanoid robot Talos in gaits that correspond to different states of the PSS guided by the SHC networks, as described in the text.

In the first task, the humanoid robot moves its forearms, while the PSS transits between states 1 and 2, as shown in Figure 4. When the PSS is in state 1, the humanoid robot is standing on its legs and has its forearms oriented downwards, as can be seen in Figure 5a.

When a vertical pulling force oriented upwards, $F_{\mathrm{up}}$, is applied to its grippers, the humanoid robot starts to lift up its forearms, and the PSS starts to transit to state 2. When state 2 is reached, the humanoid robot has its forearms oriented horizontally, as shown in Figure 5b, in the same way as humans would when they are assisted by other people that help them to move. If no forces are further applied, F=0, after a certain amount of time, the PSS transits back to state 1, and the humanoid robot puts its forearms down.

The second task comprises two movements, as presented in Figure 4. In the first movement, the humanoid robot squats, as shown in Figure 5c, while the PSS transits from state 2 to state 3. In the second movement, the humanoid robot stands up with its forearms oriented horizontally, while the PSS returns to state 2. The transition of the PSS from state 2 to state 3 is triggered by a vertical pushing force oriented downwards, $F_{\mathrm{down}}$, applied on the grippers of the humanoid robot. If no forces are further applied when the PSS reaches state 3, and the humanoid robot is in the squatting position, both the PSS and the humanoid robot remain in this state forever. On the other hand, the transition from state 3 back to state 2 is triggered by a vertical pulling force oriented upwards, $F_{\mathrm{up}}$, and the humanoid robot stands up with its forearms oriented horizontally. If no forces are further applied when state 2 is reached after a certain amount of time, the PSS transits back to state 1, and the humanoid robot puts its forearms down again and, therefore, performs the movement of the first task.

In the third task, the humanoid robot walks forwards and backwards, and, therefore, multiple movements are employed, while the PSS transits between states 2, 4, 5, 6, 7, and 8, as schematically depicted in Figure 4. The initial humanoid robot configuration of the third task is the standing up position with its forearms oriented horizontally, corresponding to state 2 of the PSS. If a pulling force oriented horizontally, $F_{\mathrm{pull}}$, is applied on the grippers of the humanoid robot, the PSS transits from state 2 to state 4, and the humanoid robot performs the first step forwards with its left leg, seen in Figure 5d. For the transitions between states 4–8, the following principles hold. If a horizontal pulling force, $F_{\mathrm{pull}}$, is applied on the grippers of the humanoid robot, the PSS transits to the state that makes the humanoid robot perform a step forwards. If a horizontal pushing force is applied on the grippers of the humanoid robot, $F_{\mathrm{push}}$, the PSS transits to the state that makes the humanoid robot perform a step backwards. Figure 5e shows the humanoid robot with its right leg forwards or left leg backward, which correspond to states 5 or 8 of the PSS, while Figure 5f shows the humanoid robot with its left leg forwards or right leg backward, corresponding to states 7 or 6 of the PSS, respectively. However, if no force is applied when the PSS is in any of the states 4–8, after some time, the PSS returns to state 2, and the humanoid robot returns to the corresponding standing position with its forearms oriented horizontally. Still, if no force is applied to the humanoid robot, after some time, the PSS transits back to state 1, and the humanoid robot puts its forearms down, which is the movement of the first task.

#### 2.3.2. Motion Synthesis

The movements of the humanoid robot were synthesized in different ways. The movement of the first task, where the humanoid robot lifts up its forearms, is a simple movement as only the angles of the elbows were modified. All the movements of the second and the third task are much more complex as multiple joint angles need to be simultaneously modified, each with a different non-constant velocity. Furthermore, throughout every movement from the initial to the final position, the stability of the humanoid robot had to be granted.

The movement of the first task, which corresponds to the PSS transition from state 1 to state 2, was created in the Gazebo simulator [64], where the humanoid robot was first positioned in its default standing position of the default controllers. Guided by a Matlab script, by specifying only the final angles of the elbows, the humanoid robot moved its forearms to the final position.

**Figure 6:**
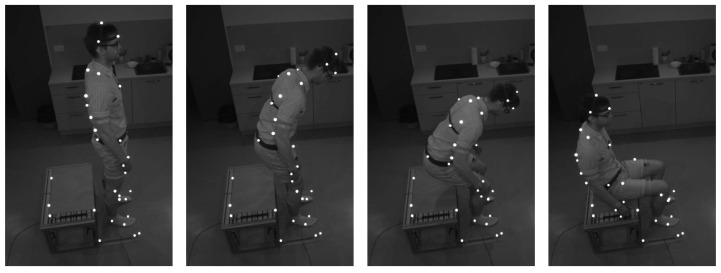
Photo sequence of a person sitting down on a bench while being recorded by the motion capture system.

The squatting and the standing up motions of the second task, which correspond to the transitions from state 2 to state 3 and back to state 2 of the PSS, were obtained by applying human motion, which was recorded with a motion capture system as shown in Figure 6, to the humanoid robot.

The movements for the walking steps of the third task, which represent the transitions between states 2 and 4–8 of the PSS, were obtained by a humanoid robot walking simulation in the Gazebo simulator. Here the humanoid robot was first positioned in the standing-up pose with its forearms oriented horizontally, corresponding to state 2 of the PSS. Then, with the integrated ros service command do_step of the walking controller, which is a ros control plugin and can run with the default controllers, the humanoid robot performed the first step forward with its left leg, two more steps forward with both legs, and a half step back with its right leg to the initial position with both legs positioned in parallel one next to the other. All the other steps were obtained as the reverse of these recorded steps.

#### 2.3.3. Motion Parametrization

During every movement of the humanoid robot in the Gazebo simulator, all its joint angles were recorded and later parametrized using the Gaussian radial basis functions (GRBF) method [65]. With this parametrization, the time dependence of a joint angle during a certain movement is transformed to phase dependence, τ∈[0,1], while the recorded angles are expressed with a combination of *N* Gaussian functions

(11) $$\Psi_{i}\left(\tau \right)=\exp\left(-\frac{{\left(\tau -c_{i}\right)}^{2}}{2\sigma_{i}^{2}}\right)$$

as

(12) $$\Phi_{\mathrm{GRBF}}\left(\tau \right)=\frac{\sum_{i}\omega_{i}\Psi_{i}\left(\tau \right)}{\sum_{i}\Psi_{i}\left(\tau \right)}\;,$$

where $c_{i}$ and $\sigma_{i}$ are the centers of the peak and the standard deviation of the *i*-th Gaussian function, respectively, $\omega_{i}$ is the weight for the *i*-th Gaussian function, while *i* goes from 1 to an arbitrary *N*.

**Figure 7:**
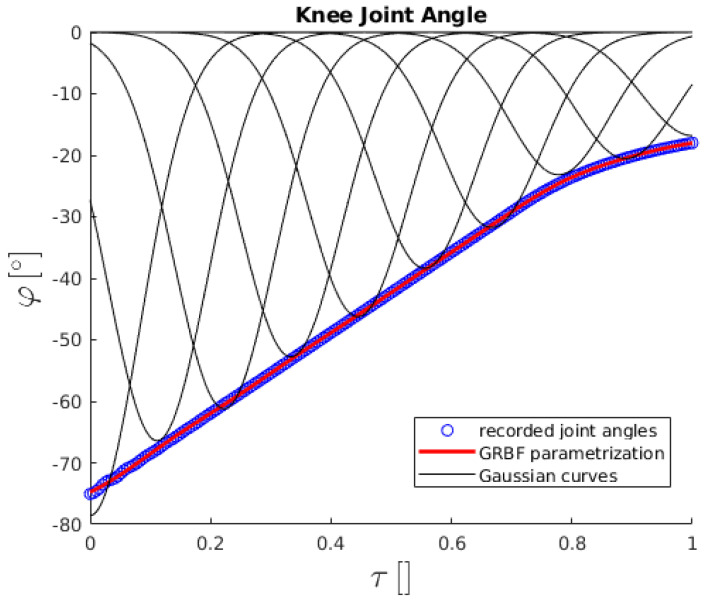
The knee joint angle of the humanoid robot during the standing-up motion as a function of phase τ. The blue empty circles are the recorded knee joint angles, the black curves are the Gaussian functions, and the red curve is the GRBF parametrization from Equation (12) of the knee joint angle, composed from the Gaussian curves from Equation (11).

An example of the GRBF method is shown in Figure 7 for the knee joint angle of the humanoid robot during the standing-up motion. As can be seen, the Gaussian functions sum up to parametrize the values of the knee joint angle throughout the whole movement. Moreover, if *N* is chosen correctly, the GRBF method cancels out the oscillations of the angle that can be attributed to the vibrations of the robot.

With such a parametrization of all joint angles, their values can be obtained for every τ. Furthermore, the phase of the transition of the PSS guided by the SHC networks can be coupled to the phase of the GRBF parametrization of the robot joint angles. This way, the phase of the PSS can directly determine the robot configuration and guide its motion.

## 3. Results

### Application of the PSS Guided by the SHC Networks to the Humanoid Robot

The dynamical PSS guided by the SHC networks, described in Section 2.1, was applied to guide the motion of the humanoid robot, as described in Section 2.3.

To enable the transitions between the PSS states as presented in the sketch in Figure 4, the state transition matrix was set to

(13) $$T=\left[\begin{array}{cccccccc} 0 & 1 & 0 & 0 & 0 & 0 & 0 & 0\\ 1 & 0 & 1 & 1 & 1 & 1 & 1 & 1\\ 0 & 1 & 0 & 0 & 0 & 0 & 0 & 0\\ 0 & 1 & 0 & 0 & 0 & 0 & 0 & 0\\ 0 & 0 & 0 & 1 & 0 & 1 & 1 & 0\\ 0 & 0 & 0 & 0 & 1 & 0 & 0 & 1\\ 0 & 0 & 0 & 0 & 1 & 0 & 0 & 1\\ 0 & 0 & 0 & 1 & 0 & 1 & 1 & 0 \end{array}\right]\;.$$

The matrix G, the vectors α, β, and ν, along with the step size dt and the parameter γ of the PSS were all set to the same values as for the three-state system presented in Section 2.2. The values of the bias matrix were set for the transition from the state *i* to the state *j* in the following way: for the transition that was blocked, $B_{ji}=-10^{-3}$, for the transition that was triggered, $B_{ji}=10^{-1}$, for the transition that was triggered after some time, $B_{ji}=10^{-3}$, while for the transition that was reversed, $B_{ij}=10$.

A transition was triggered if forces in an absolute value larger than 15N were applied in the horizontal or in the vertical direction to both grippers of the humanoid robot, and if forces applied in this direction could trigger a transition from the current state of the PSS. However, when pulling or pushing the humanoid robot for its grippers, forces were always applied in both the vertical and the horizontal direction simultaneously. The considered direction was, therefore, chosen as the direction in which the sum of the absolute values of the forces in both grippers was larger. On the other hand, if the PSS was in state 2 or in states 4–8 and no forces were exerted on the humanoid robot, the transition to state 1 or to state 2, respectively, was automatically triggered after some time. Moreover, if during a transition, forces were applied in the opposite direction of the forces that triggered the transition, the transition was reversed, and the PSS returned to the initial state. It should be emphasized that after a transition was triggered and if it was not reversed, the PSS reached the final state also if no forces applied in the same direction as the ones that triggered the transition, were exerted. However, if during a transition, forces were applied in the same direction as the ones that triggered it, the velocity of the transition was increased.

**Figure 8:**
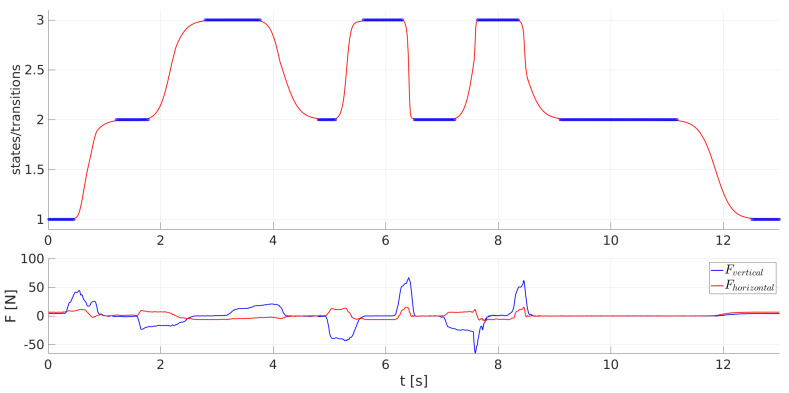
Transitions of the PSS between states 1–3 as a function of time with the forces exerted on the grippers of the humanoid robot.

The velocities of the transitions between different PSS states and, therefore, between different configurations of the humanoid robot were modulated according to Equation (9) by the parameter $A=-1.5+10^{-2}{F_{\mathrm{grip}}}^{1.4}$, where $F_{\mathrm{grip}}=0.5\left(F_{L}+F_{R}\right)$, with $F_{L}$ and $F_{R}$ being the forces applied to the left and right gripper of the humanoid robot, respectively, in the direction that triggered the current movement. A demonstration of how forces influence the velocities of PSS transitions is shown in Figure 8, where in the beginning, the PSS transits from state 1, in which the humanoid robot is standing with its forearms oriented downwards, to state 2, in which the humanoid robot lifts its forearms in the horizontal position. Further on, the PSS transits between states 2 and 3, while the humanoid robot transits between the squatting and the standing-up position. In the end, the PSS returns to state 1, and the humanoid robot puts its forearms back down. The transitions between states 2 and 3 were triggered with different forces, and, therefore, the transitions occurred at different velocities. The first two transitions from state 2 to state 3 and back to state 2 were triggered at times 1.8 s and 3.8 s, respectively, by vertical forces with absolute values of approx. 20 N, and, therefore, both transitions lasted approximately 1 s. The third transition, from state 2 to state 3, was triggered at 5.1 s by vertical forces of approx. −40 N, while the transition lasted approx. 0.5 s. The vertical force that triggered the fourth transition from state 3 to state 2, starting at 6.3 s, was approx. 60 N, while the transition lasted approx. 0.2 s. The last two transitions between states 2 and 3 had varying velocities as the forces acting on the grippers of the humanoid robot had varying magnitudes throughout the transition. The vertical force triggering the first of the last two transitions at 7.2 s was approx. −25 N and increased to −65 N, which caused the acceleration of the transition velocity. On the other hand, the force triggering the last transition from state 3 to state 2 at 8.4 s was the first of approximately 55 N, but later ceased, and, therefore, the transition of the PSS started with an elevated velocity but later decreased to its default value.

**Figure 9:**
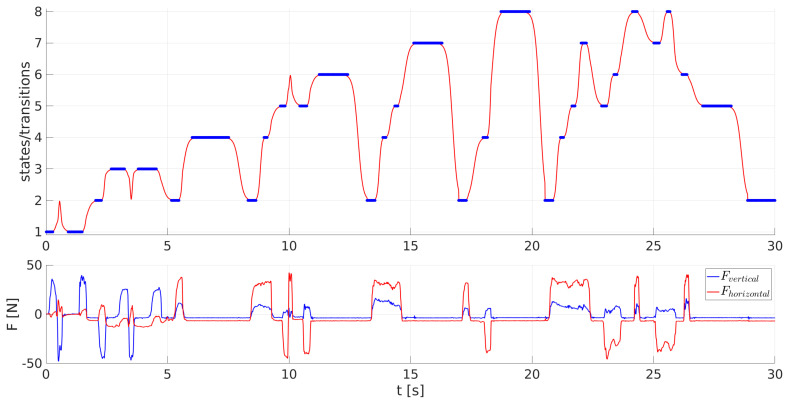
Transitions of the PSS between states 1–8 as a function of time with the forces exerted on the grippers of the humanoid robot.

A demonstration of all possible PSS transitions, along with the horizontal and vertical forces exerted on the grippers of the humanoid robot, is shown in Figure 9.

In the beginning, the 1st task is demonstrated as initially, the PSS was in state 1, and the humanoid robot was standing with its forearms oriented downwards. After applying a pulling vertical force oriented upwards to the humanoid robot, the system started at 0.3 s to transit to state 2, while the humanoid robot started lifting its forearms. However, during the transition, a pushing vertical force was applied to the grippers of the humanoid robot, and, therefore, the PSS returned to state 1, and the humanoid robot put its forearms back down. Later on, the system was pushed to state 2 by reapplying a pulling vertical force oriented upwards, and at time 1.5 s, the humanoid robot started to lift its forearms in a horizontal position.

To continue, the 2nd task is presented. Pushing vertical forces oriented downwards were applied on the humanoid robot standing with its forearms oriented horizontally, and at 2.3 s, the PSS started to transit to state 3 while the humanoid robot squatted. When the humanoid robot started to stand up at 3.2 s, as pulling forces were applied, an opposite force was exerted on its grippers, so the PSS transited back to state 3, and the humanoid robot returned to the squatting position. At 4.5 s, pulling forces were again applied, so the PSS transited back to state 2, and the humanoid robot stood up with its forearms oriented horizontally.

Finally, from 5.5 s on, the transitions of the 3rd task occurred, and the humanoid robot performed walking movements. As it can be seen in Figure 9, pulling forces exerted in the horizontal direction caused such transitions of the PSS that made the humanoid robot perform steps forward, while the transitions of the PSS caused by pushing forces in the horizontal direction made the humanoid robot perform steps backward. A transition worth emphasizing was the one at time 9.8 s when the PSS was in state 5, and a pushing horizontal force started its transition to state 6, while the humanoid robot started moving back with its right leg positioned forwards. During this transition, a pulling horizontal force was exerted on the humanoid robot, so the PSS reversed its transition back to state 5, and the humanoid robot returned its right leg forwards. However, when no forces were exerted on the humanoid robot and the PSS was in states 4–8, a spontaneous transition to state 2 occurred. This happened at times 7.5 s, 12.4 s, 16.3 s, 19.9 s, and 28.2 s and made the humanoid robot put its legs one next to the other while keeping its forearms in the horizontal position.

Each time a different force was applied to the grippers of the humanoid robot. The velocities of the transitions that are triggered by these forces were, therefore, different for every transition. Moreover, as the applied forces were modified and also ceased during various transitions, the transition velocities were also changing during the courses of the transitions. See the Supplementary Materials for a sample video of how the humanoid robot is guided.

## 4. Discussion

Robots are being employed in many different areas to assist humans with various tasks. Humanoid robots that can mimic human behavior and can perform human-like motions are due to the human–robot skill transfer methods and techniques of special interest. However, understanding human intentions and performing safe and at the same time efficient cooperation is very challenging. For this reason, the area of human–robot collaboration is still in its early days, while cooperation between humans and robots is still very limited. Nevertheless, this area of robotics has undergone tremendous advance in the last decades, and engaging experts from various fields of science are evolving at an incredible speed.

In this paper, human–robot collaboration is addressed with the application of a dynamical PSS guided by the SHC networks to the humanoid robot. Such a dynamical model can smoothly transit between its states with varying velocity, while each transition can be reversed, and the system can return to its initial state. As every PSS transition has its phase that increases from 0 when the PSS is in its initial state, to 1 when the PSS reaches its final state, it was coupled to the robot that moves from its initial to its final configuration. This way, the PSS controls the robot gate throughout the movement. The transitions between PSS states can occur automatically or can be triggered by certain events or conditions that must be fulfilled, depending on the executed task.

The dynamical PSS presented in this paper consists of eight states linked to eight different configurations of the humanoid robot. Some of the transitions between the discussed PSS states are triggered by the forces applied to the grippers of the humanoid robot, while some of them occur automatically if no forces are applied. Such an approach allows humans to guide a humanoid robot in the same way as they would guide another person when assisting him/her in moving. Furthermore, the larger the applied forces, the faster the movement of the robot, and vice versa. This allows humans to dynamically control the motion of the humanoid robot and even reverse its motion toward its initial state if forces in the opposite direction are applied.

The largest advantage of using a PSS controlled by the SHC networks for guiding a robot is its simplicity of application. All the possible transitions are determined by a single matrix T, while a specific transition is triggered by the forces applied in a certain direction, which determines the matrix B. In the model presented in this paper, vertical forces make the humanoid robot squat and stand up, while horizontal forces make it walk forwards and backward. However, the PSS used to control the motion presented in this paper could be extended to many other states and movements of the robot.

The movements of the humanoid robot presented in this paper were pre-determined and parameterized using the GRBF method. The squatting and standing-up movements were obtained by filming a person and applying his reconstructed joint angles to the humanoid robot, while the walking movements were obtained with a computer simulation in the Gazebo simulator. The use of pre-defined motion is also the largest limitation of the presented implementation of the discussed dynamical PSS, as the robot is limited to the pre-recorded movements. Nevertheless, the presented approach provides an important step towards human–robot collaboration and interaction as the robot must understand human intentions, expressed with the forces applied on its grippers, to perform the desired movement. Moreover, for the modulation of the motion velocity, a further interpretation of the human will is done in terms of considering the magnitude of the applied forces. With such an understanding of human intentions, the next step could be to adapt and improve the robot motion with the application of learning algorithms and to further synthesize new robot movements according to the needs of the human collaborator.

In this paper, two sample tasks, squatting and walking, were performed by the humanoid robot in collaboration with a human, who was leading the robot by pulling and pushing it in the horizontal and vertical direction for its grippers. However, the range of robot movements, along with the guiding mechanisms and signals that the robot can understand from the human leader, can increase by an undetermined amount. This way human–robot collaboration can evolve and expand to many different areas while robots can learn to cooperate in different ways and help humans to perform a numberless amount of tasks. Furthermore, with the inclusion of the learning algorithms for the improvement of the robot motion and execution of different tasks, the human–robot collaboration could become even smoother and faster, while the robots could take a major role in the collaborative tasks.

## 5. Conclusions

With rising costs and decreasing availability of human labor, the demand for robots and robotic assistants increases on daily basis in many different areas. However, despite the enormous advance the field of assistive robotics has undergone in the last decades, the cooperation between humans and robots is still very limited. For this reason, new methods for understanding human intentions and controlling assistive robots, while providing a safe environment, need to be explored and developed.

In this paper, a promising approach for realizing human–robot collaboration is presented. The motion of the humanoid robot is controlled by a dynamical PSS consisting of 8 states, guided by the SCH networks. Such a mathematical model is easy to implement and simple to control, as only a handful of matrices and parameters need to be determined and modified throughout the evolution of the model. As the transitions between the PSS states are triggered and modulated by the directions and amplitudes of the forces exerted on the grippers of the humanoid robot by a human leader, the robot must understand human intentions to execute the desired task. This is the reason why the presented strategy represents a big step towards human–robot collaboration, as it combines the understanding of human intentions with the execution of the desired tasks in the desired way. However, as there is an infinite amount of tasks that a robot can perform, such a dynamical PSS can be expanded and upgraded for collaborative robots to assist humans in many different areas such as industry, healthcare, medicine, hospitality and tourism, home assistance, and many more.

## Data Availability

All the data presented in this paper can be provided by the authors of this paper upon request.

## Supplementary Materials

The following supporting information can be downloaded at: https://www.mdpi.com/article/10.3390/s23031396/s1, A video of the humanoid robot Talos guided by a person and performing the motions presented in this paper.

## Abbreviations

The following abbreviations are used in this manuscript:

PSS	Phase State System
SHC	Stable Heteroclinic Channel
GRBF	Gaussian Radial Basis Functions
